# Cousins at work: How combining medical with optical imaging enhances *in vivo* cell tracking

**DOI:** 10.1016/j.biocel.2018.06.008

**Published:** 2018-09

**Authors:** Alessia Volpe, Ewelina Kurtys, Gilbert O. Fruhwirth

**Affiliations:** Department of Imaging Chemistry and Biology, School of Biomedical Engineering and Imaging Sciences, King’s College London, SE1 7EH, London, UK

**Keywords:** BLI, bioluminescence imaging, CEST, chemical exchange saturation transfer, CLI, Cerenkov luminescence imaging, CM, confocal fluorescence microscopy, CT, X-ray computed tomography, FLI/FRI, fluorescence imaging/fluorescence reflectance imaging, FMT, fluorescence mediated tomography, HF, high-frequency, IVM, intravital microscopy, MRI, magnetic resonance imaging, MSOT, multispectral optoacoustic tomography, NIR, near-infrared spectrum, OCT, optical coherence tomography, OPT, optical projection tomography, PAT, photoacoustic tomography, RSOM, high-resolution raster scanning optoacoustic mesoscopy, PET, positron emission tomography, SPECT, single photon computed emission tomography, SRM, super-resolution microscopy (a group of various technologies including but not limited to photoactivated localisation microscopy (PALM), various stochastic optical reconstruction microscopy (STORM) techniques, stimulated emission depletion microscopy (STED), and ground state depletion individual molecule return (GSDIM)), TPM, tow-photon excitation microscopy, US, ultrasound imaging including Doppler and high-frequency ultrasound techniques, VIS, visible light spectrum, Cancer metastasis, Cell therapy, Microscopy, Reporter genes, Whole-body imaging

## Abstract

Microscopy and medical imaging are related in their exploitation of electromagnetic waves, but were developed to satisfy differing needs, namely to observe small objects or to look inside subjects/objects, respectively. Together, these techniques can help elucidate complex biological processes and better understand health and disease. A current major challenge is to delineate mechanisms governing cell migration and tissue invasion in organismal development, the immune system and in human diseases such as cancer where the spatiotemporal tracking of small cell numbers in live animal models is extremely challenging.

Multi-modal multi-scale *in vivo* cell tracking integrates medical and optical imaging. Fuelled by basic research in cancer biology and cell-based therapeutics, it has been enabled by technological advances providing enhanced resolution, sensitivity and multiplexing capabilities. Here, we review which imaging modalities have been successfully used for *in vivo* cell tracking and how this challenging task has benefitted from combining macroscopic with microscopic techniques.

## 1 Introduction

**Fig. 1:**
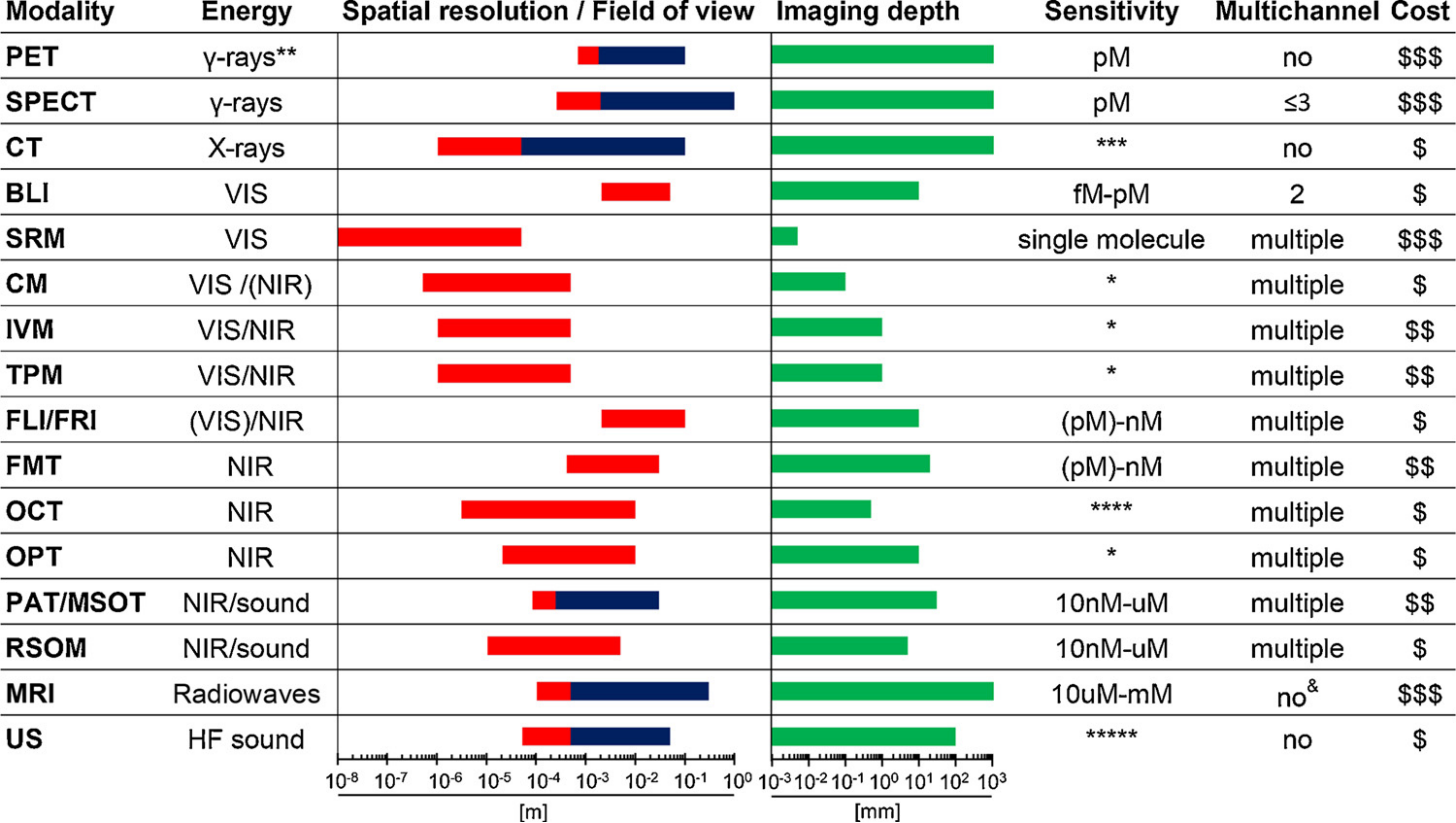
Macroscopic and microscopic imaging modalities. Imaging modalities are ordered according to the electromagnetic spectrum they exploit for imaging (top: high energy; bottom: low energy). Routinely achievable spatial resolution (left end) and fields of view (right end) are shown in red. Where bars are blue they overlap red bars and indicate the same parameters but achievable with instruments used routinely in the clinic. Imaging depth is shown in green alongside sensitivity ranges. Instrument cost estimations are classified as ($) <125,000 $, ($$) 125–300,000 $ and ($$$) >300,000 $. * Fluorophore detection can suffer from photobleaching by excitation light. ** Generated by positron annihilation (511 keV). *** Contrast agents sometimes used to obtain different anatomical/functional information. **** In ‘emission mode’ comparable to other fluorescence modalities (∼nM). ***** Highly dependent on contrast agent. ^&^ Multichannel MRI imaging has been shown to be feasible (Zabow et al., 2008). (For interpretation of the references to colour in this figure legend, the reader is referred to the web version of this article).

Two major discoveries, one enabling observation of smaller objects and the other allowing to look inside subjects/objects, significantly boosted biological/biomedical research. The first compound microscope was invented by Hans and Zaccharias Jansen in the late 16^th^ century, which triggered later microscopy development that in turn enabled the direct observation of atoms, single molecules and single-/multi-cellular organisms including their dynamics. The second transformation was Wilhelm Roentgen’s discovery of X-rays in 1895, which enabled investigations of inner subject/object structures in a non-invasive way (genetic effects of radiation were only recognized later) and founded medical imaging. Both microscopy and medical imaging rely on the interaction of biological matter with electromagnetic waves, but medical imaging employs a wider range than microscopy including α/β/γ-ray-emitting radioisotopes, X-rays, visible (VIS)/near-infrared (NIR) light, radio waves and ultrasound (Fig. 1). Medical imaging revolutionized the diagnosis and treatment of human disease by providing anatomical, physiological and molecular information (Mankoff, 2007). Imaging modalities differ in their capabilities and limitations (Fig. 1), hence combination technologies were introduced to exploit them best (‘multi-modal imaging’). For example, positron emission tomography (PET) offers best-in-class sensitivity and absolute quantification but only at millimetre resolution and was combined with modalities providing higher resolution such as computed tomography (CT) (Basu et al., 2014) or magnetic resonance imaging (MRI) (Catana, 2017). How medical imaging can be used to develop biomarkers providing diagnostic, prognostic, predictive, and treatment monitoring information was recently standardized (O’Connor et al., 2017). Photoacoustic tomography (PAT) and Cerenkov luminescence imaging (CLI) are special in that they both rely on electromagnetic waves from different parts of the spectrum for imaging. PAT delivers NIR laser pulses into biological tissues with the latter absorbing and converting some of the laser pulse energy into heat, leading to transient thermoelastic expansion and thus wideband ultrasonic emission (Ntziachristos et al., 2005; Wang and Yao, 2016). CLI relies on the collection of light produced by charged particles traversing through biological tissue with a velocity greater than the phase velocity of light in that medium (Ciarrocchi and Belcari, 2017). Brightfield microscopy and, less frequently, fluorescence microscopy are routine techniques providing confirmatory pathology information obtained from biopsied tissues. Recently, automated multiplex fluorescence histopathology (Mansfield et al., 2008; Stack et al., 2014) has enabled rigorous tissue profiling, *e.g.* immune infiltration in tumour tissues (Galon et al., 2014).

Here, we review which imaging modalities have been successfully used for *in vivo* cell tracking and how this challenging task benefitted from combining macroscopic with microscopic techniques. For detailed information on the instrumentation of individual imaging technologies and their use, we provide references to recent specialist literature.

## 2 The need for *in vivo* cell tracking in cancer research

A major challenge in cancer research is to better understand the mechanisms governing cell migration and tissue invasion. A plethora of different models including animal tumour models are used for this purpose. It remains extremely challenging to reliably quantify the *in vivo* distribution, relocalisation, and viability of cancer cells in animal tumour models, which are sufficiently large to be optically opaque. For example, the spatiotemporal quantification of cancer cell spread in mouse models of metastasis is a needle-in-a-haystack task. Traditionally, in preclinical cancer research one target organ of metastasis was chosen, large animal cohorts were sacrificed at different time points to overcome inter-animal variability and these approaches were paired with microscopic or flow cytometric analyses in target tissues as read-outs. Whole-body imaging can (i) inform on *in vivo* cell distribution, for example, visualize unexpected metastatic sites; (ii) provide quantitative data, *e.g.* live tumour volumes/metastatic burden and extent of cell therapy on-site residence over time; (iii) provide cell viability data; (iv) reduce inter-subject variability as serial imaging of the same subjects provides statistically better paired data; and (v) can minimize animal usage during preclinical development. Similarly, when developing anti-cancer drugs, it is important to establish targeting efficiency, pharmacokinetics and pharmacodynamics, whether there is spatial heterogeneity to the delivery, and if drug presence is related to therapeutic efficacy. Again, this can be achieved by combining preclinical whole-body cancer cell tracking with conventional molecular imaging of drugs, for example, by image-based quantification of the extent a labelled drug reaches *in vivo* traceable cancer cells and whether the drug is delivered to all primary/secondary lesions.

Another area where *in vivo* cell tracking is an emerging valuable tool is the development and clinical translation of cell-based therapies. Unlike conventional chemotherapeutics or targeted therapies, they cannot be considered as ‘fire-and-forget’ weapons in the battle against cancer as they are live cell products, but little is known about their *in vivo* distribution and fate both preclinically and clinically. In 2017, the FDA approved the first clinical products, tisagenlecleucel and axicabtagene ciloleucel, which are autologous CD19b-targeted chimeric antigen receptor T-cell (CAR-T) immunotherapies for the treatment of certain blood cancers (B-cell lymphomas; (USFood&DrugAdministration, 2017a,b)). CAR-T immunotherapies have the potential to be curative, but not all patients respond and sometimes the effects are only temporary (Maude et al., 2018; Neelapu et al., 2017; Schuster et al., 2017). CAR-T are also associated with severe/life-threatening side-effects and fatalities during trials (Linette et al., 2013; Saudemont et al., 2018). Moreover, cellular immunotherapeutics for treating solid tumours are at the clinical trial stages but not yet routinely available to patients. Traditional approaches in preclinical cell therapy development rely on dose escalation with toxicity evaluation, tumorigenicity tests, and qPCR-based persistence determination. However, clinical trials are still performed without knowledge about the *in vivo* distribution and fate of the administered therapeutic cells, making it impossible to adequately monitor and assess their safety. Major unresolved questions in cell therapy development and use both preclinically and clinically are: (i) the whole-body distribution of therapeutic cells; (ii) their potential for re-location during treatment and the kinetics of this process; (iii) whether on-target off-site toxicities occur; (iv) how long the administered cells survive; and (v) which biomarkers are best suited to predict and monitor cell therapy efficacy. Whole-body imaging-based *in vivo* cell tracking can inform on many of these aspects in a truly non-invasive manner.

## 3 Rendering cells traceable *in vivo*

*In vivo* cell tracking exploits molecular imaging mechanisms but differs from conventional molecular imaging as contrast agents or contrast-forming features are added to the cells before their administration into subjects. On some occasions, features that can be exploited for generating contrast are intrinsic, for example, when cancer cells express molecules that show low or no expression in other tissues. Under these circumstances conventional molecular imaging offers tracking possibilities both preclinically and clinically (*e.g.* sodium iodide symporter (NIS) in thyroid metastases (Kogai and Brent, 2012; Portulano et al., 2014), glutamate carboxypeptidase 2 (PSMA) in prostate cancer (Oliveira et al., 2017; Perera et al., 2016), carcinoembryonic antigen in colorectal cancer (Tiernan et al., 2013), or melanin in melanomas (Tsao et al., 2012)). However, in most cases contrast agents or contrast-forming features must be introduced to the cells of interest, and, crucially, this must be done with the experimental design in mind (technology, tracking time, tracking interval, preclinical/clinical setting).

Labels can be introduced into cells *via* two fundamentally different methodologies. So-called ‘direct cell labelling’ employs ready-to-use contrast agents (*e.g.* organic fluorophores, quantum dots, iron oxide nanoparticles, ^19^F-fluorinated contrast agents, chelated radiometals *etc.*), which are introduced into cells either due to the contrast agents being cell permeant, or through assisted uptake (*e.g.* by transfection or internalisation) (Kircher et al., 2011). The alternative is ‘indirect cell labelling’, whereby a genetically encoded reporter is ectopically introduced to the cells mostly by viral transduction to ensure genomic integration and long-term expression. In some cases, episomal plasmids (*e.g.* delivered using transfection or electroporation to deliver the DNA) can also be useful. Lately, gene editing approaches have been reported for reporter insertion, which are have advantages over viral transduction as they offer precise control over the genomic site of reporter insertion (Bressan et al., 2017). Contrast formation relies on either (a) label uptake by reporters that are transporters, (b) label binding to cell surface-expressed reporters, or (c) expression of contrast-forming proteins (*e.g.* fluorescent proteins, luciferases). All these indirect mechanisms find utility in reporter gene applications, which are used to image intracellular molecular events or, as discussed here, to perform *in vivo* cell tracking.

Reporter genes (Table 1) have critical advantages over direct labelling for cell tracking. First, the observation period is independent of the contrast agent, for example, not affected by the half-lives of a radioisotope. Second, genetic encoding avoids label dilution phenomena, which are particularly limiting observation times in the case of fast growing cells (*e.g.* cancer cells or expanding T cells). Third, genetic encoding circumvents complex cell labelling procedures and potential associated cell damage/toxicities. A drawback of the indirect cell labelling approach is that it requires genetic engineering. However, this is neither a concern for preclinical experimentation nor for cell therapies already reliant on it (*e.g.* CART) (Saudemont et al., 2018). A caveat exists in the potential for immune system activity against reporters as cells expressing foreign reporters can be detected, attacked, and cleared by an intact immune system. This may best be overcome by using host reporter proteins that are normally endogenously expressed in the organism of interest. Importantly, these host reporters should be endogenously expressed in only a limited number of host tissues, to exclude interference with the experimental goals, and ideally at low levels to ensure favourable contrast.

**Table 1:** Reporter genes and corresponding imaging tracers and substrates.

Reporter type	Reporter name	Imaging tracer / substrate	Properties	Limitations	Ref.
Cell surface receptor	Human somato-statin receptor type 2 (hSSTr2)	**PET:**^68^Ga-DOTATOC, ^68^Ga-DOTATATE; **SPECT:**^111^In-DOTA-BASS; (best tracers selected here).	G-protein-coupled receptor; several tracers cross the BBB.	Endogenous expression in brain, adrenal glands, kidneys, spleen, stomach and many tumours (*i.e.* SCLC, pituitary, endocrine, pancreatic, paraganglioma, medullary thyroid carcinoma, pheochromocytoma); tracers may cause cell signalling and change proliferation.	(Chaudhuri et al., 2001; Rogers et al., 1999, 2000; Zinn et al., 2000)
Cell surface receptor	Dopamin receptor (D_2_R)-	**PET:** [^18^F]FESP, [^11^C]Raclopride, [^11^C]N-methylspiperone.	G-protein-coupled receptor; tracers cross BBB.	Slow clearance of [^18^F]FESP; high background in the pituitary gland and striatum due to endogenous expression.	(Hwang et al., 2007;Liang et al., 2001; MacLaren et al., 1999; Satyamurthy et al., 1990)
Cell surface receptor	Transferrin receptor (TfR)	**MRI:** Transferrin-conjugated SPIO.		Transferrin-conjugated SPIO particles are internalized by cells ectopically expressing TfR.	(Weissleder et al., 2000)
Cell surface-expressed antigen	Human Carcinoembryonic antigen (hCEA)*	**PET:**^124^I-anti-CEA scFv-Fc H310 A antibody fragment, [^18^F]FB-T84.66 diabody; **SPECT:**^99m^Tc-anti-CEA Fab’ (FDA approved), ^111^In-ZCE-025, ^111^In-anti-CEA F023C5i.	Overexpressed in pancreatic, gastric, colorectal and medullary thyroid cancers.	CEA not expressed in healthy adult human cells, except for colon lumen; tracers do not cross BBB.	(Griffin et al., 1991; Hammarstrom, 1999; Hong et al., 2008; Kenanova et al., 2009)
Cell surface protein	Glutamate carboxypeptidase 2* (PSMA)	**PET:** [^18^F]DCFPyL, [^18^F]DCFBC; **SPECT:** [^125^I]DCFPyL; anti-PSMA antibodies can be flexibly labelled, e.g J951-IR800.		Background signal in kidneys; tracers do not cross BBB.	(Castanares et al., 2014)
Transporter	Sodium iodide sym-porter (NIS) [human, mouse, rat]	**PET:**^124^I^−^, [^18^F]BF_4_^−^, [^18^F]SO_3_F^−^, [^18^F]PF_6_^−^; **SPECT:**^99m^TcO_4_^−^; ^123^I^−^.	Symports sodium ions.	Endogenously expressed in lthyroid, stomach, lacrimal, salivary and lactating mammary glands, small intestine, choroid plexus and testicles; tracers do not cross BBB.	(Dai et al., 1996; Jauregui-Osoro et al., 2010; Jiang et al., 2018; Khoshnevisan et al., 2017, 2016)
Transporter	Norepinephrin transpo-rter (NET)	**PET:** [^124^I]MIBG, [^11^C]hydroxyephedrine; **SPECT:** [^123^I]MIBG.		Endogenously expressed in organs with sympathetic innervation (heart, brain), tracers do not cross BBB.	(Moroz et al., 2007)
Transporter	Dopamin transporter (DAT)	**PET:** [^11^C]CFT, [^11^C]PE2I, [^18^F]FP-CIT; **SPECT:**^123^I-β-CIT, ^123^I-FP-CIT, ^123^I-Ioflupane, ^99m^TRODAT.	NaCl-dependent; tracers cross BBB.	Few data in public domain.	Patent: (Martin Pulé (London), 2015)
Artificial cell surface molecule	Anti-PEG Fab fragment*	**PET:**^124^I-PEG-SHPP; **MRI:** SPIO-PEG; **Fluorescence:***e.g.* NIR797-PEG.	Some tracers cross BBB; PEG is non-toxic and FDA approved.	Iodine tracers bear risk of deiodination.	(Chuang et al., 2010)
Artificial protein	Lysine-rich protein	**MRI:** Chemical exchange saturation transfer (CEST).	Frequency-selective contrast.		(Farrar et al., 2015; Gilad et al., 2007)
Enzyme	HSV1-*tk* and mutants-	**PET:** [^124^I]FIAU, [^18^F]FEAU, [^18^F]FHBG.	Kinase causing cellular tracer trapping; suicide gene property.	Tracers do not cross the BBB; high activity in organs involved in clearance.	(Tjuvajev et al., 1995)
Enzyme	hmtk2/hΔTK2	**PET:** [^124^I]FIAU, [^18^F]FEAU, [^18^F]FMAU (hTK2-N93D/L109 F).	Kinase causing cellular tracer trapping.	Tracers do not cross the BBB.	(Ponomarev et al., 2007)
Enzyme	hdCK	**PET:** [^124^I]FIAU, [^18^F]FEAU.	Kinase causing cellular tracer trapping.	Tracers do not cross the BBB.	(Lee et al., 2017; Likar et al., 2010)
Enzyme	β-galacto-sidase	**PET:** 2-(4-[^123^I]iodophenyl)ethyl-1-thio-*β-D*-galactopyranoside, 3-(2’-[^18^F]fluoroethoxy)-2-nitrophenyl-*β-D*-galactopyranoside, 3-[^11^C]methoxy-2-nitrophenyl-*β-D*-galactopyranoside; **SPECT:** 5-[^125^I]iodoindol-3-yl-β-D-galactopyranoside; **PAT:** 4-chloro-3-bromoindole-galactose (X-gal); **MRI:** EgadMe.	Glycoside hydrolase.	Cellular toxicity may change with substrates.	(Li et al., 2007; Liu and Mason, 2010; Louie et al., 2000)
Enzyme	Tyrosinase	**PET:** [^18^F]P3BZA-melanin avid probe; **MRI:** Melanin due to ability to chelate metal ions (Fe^3+^); **PAT:** Melanin.	Copper-containing enzyme.	Low expression levels; no clinical use.	(Krumholz et al., 2011; Ponomarev et al., 2004; Weissleder et al., 1997)
Enzyme	Firefly luciferase	Luciferin and derivatives.	Substrate-dependent, (often: orange/red)	No clinical use.	(Mezzanotte et al., 2017; Ugarova, 1989)
Enzyme	Renilla luciferase	Coelenterazine	482-547 nm emission	No clinical use.	(Lorenz et al., 1991)
Enzyme	Gaussia luciferase	Coelenterazine	480-600 nm emission	No clinical use.	(Inoue et al., 2011; Tannous, 2009; Tannous et al., 2005)
Enzyme	Green Click Beetle luciferase	Luciferin, naphtyl luciferin.	Emission varies in sub-species: green (548 nm), yellow-green (565 nm), orange (594 nm) and near-infrared.	No clinical use.	(Biggley et al., 1967; Mezzanotte et al., 2014;Mezzanotte et al., 2011;Wood et al., 1989)
Monomeric fluorescent proteins (mFP)	eGFP A206K**		488(ex)/507(em) nm	No clinical use.	(Ormo et al., 1996)
	mCherry**		587/610 nm	No clinical use.	(Shaner et al., 2004)
	TagRFP**		555/584 nm	No clinical use.	(Merzlyak et al., 2007)
	mPlum**		590/649 nm; also used for PAT.	No clinical use.	(Lin et al., 2009)
	mNeptune**		600/650 nm	No clinical use.	(Kremers et al., 2009)
Fluorescent protein	E2-Crimson		611/646 nm	No clinical use, tetramer.	(Liu et al., 2013)
NIR fluorescent proteins	IFP1.4	Exogenously added biliverdin (BV)	684/708 nm	No clinical use; dimer; need for exogenous BV.	(Shcherbakova and Verkhusha, 2013; Shu et al., 2009)
	iRFP 670	Endogenous biliverdin sufficient	643/670 nm; also used for PAT.	No clinical use; dimer.	(Deliolanis et al., 2014; Filonov et al., 2011; Shcherbakova and Verkhusha, 2013)
	iRFP 713	Endogenous biliverdin sufficient	690/713 nm; also used for PAT.	No clinical use; dimer.	(Deliolanis et al., 2014; Filonov et al., 2011; Shcherbakova and Verkhusha, 2013)
Photoactivatable protein	Kaede**		518/580 nm	No clinical use.	(Ando et al., 2002)
	IrisFP**		516/580 nm	No clinical use.	(Adam et al., 2008)
Photoconvertible protein	Dendra2**		507 nm to 573 nm switch	No clinical use; switch is irreversible.	(Gurskaya et al., 2006)
Iron carrier protein	Ferritin	**MRI:** iron.		Iron is not equally distributed across the brain and therefore may cause local susceptibility shifts that are above the MRI detection limit.	(Cohen et al., 2005; Genove et al., 2005)
Gas-filled protein complex	GvpA/ GvpC	**Ultrasound:** gas vesicles generate contrast.	Reporter gene cluster required.	Not yet validated for use in mammalian cells.	(Bourdeau et al., 2018)

* Any other modality can be used provided a suitable contrast forming moiety will be attached to PEG and the CEA antibodies, respectively.

** Can be used in fusion with other reporter genes without introduction of artificial protein clustering.

## 4 Optical imaging–versatility and limitations

Selecting technology for the task of *in vivo* cell tracking is not a straightforward task. The group of optical imaging technologies overall offers the widest versatility across multiple length scales, spanning microscopy and macroscopic medical imaging (Fig. 1). Fluorescence is the only imaging modality capable to bridge the length scales (macroscopic, (sub)cellular, molecular), hence would appear most attractive for the task of *in vivo* cell tracking. For example, using one fluorescent dye, whole-body imaging and tissue microscopy data were acquired (Swirski et al., 2007). However, no single optical approach can cover all requirements for *in vivo* cell tracking despite recent technological advancements. For example, improvements in fluorescence microscopy have allowed deeper sample penetration and imaging larger specimen (*cf.* light sheet and expansion microscopy, tissue clearing (Ariel, 2017; Karagiannis and Boyden, 2018; Whitehead et al., 2017)). Moreover, intravital fluorescence microscopy offers cellular resolution in live animals, but only in very limited fields of view and in certain accessible tissues (Alieva et al., 2014; Condeelis and Segall, 2003; Entenberg et al., 2017; Entenberg et al., 2018; Pittet and Weissleder, 2011). In contrast, both fluorescence and bioluminescence whole-body imaging (BLI and FRI/FLI) offer large fields of views but suffer from poor resolution (Fig. 1) that is insufficient for *in vivo* cell tracking and are planar imaging technologies and thus unable to provide 3D or quantitative data. BLI offers orders of magnitude better sensitivities than all macroscopic fluorescence techniques and is inexpensive but requires the tissue availability of a luminescence substrate, is limited in its multiplexing capability, and confined to preclinical use (Dunlap, 2014; Jiang et al., 2016; Li et al., 2013). To obtain true 3D data a tomographic design is required. This is provided by optical projection tomography (OPT), which can be considered to be the optical analogue of X-ray computed tomography (CT). OPT operates on the micrometre to millimetre scales (Cheddad et al., 2012; Sharpe et al., 2002) thereby bridging the scale gap between BLI/FLI and microscopy. It can either provide tomographic data on light absorption or fluorescence signals, and has been used in live zebrafish (Bassi et al., 2011; McGinty et al., 2011), fruit flies (Arranz et al., 2014; Vinegoni et al., 2008) and for whole organ imaging in mice (Alanentalo et al., 2008; Gleave et al., 2012; Gupta et al., 2013). An alternative approach offering larger fields of view in the centimetre range is diffuse optical tomography or fluorescence mediated tomography (FMT), which exploits photon tissue propagation theory to allow for 3D reconstruction at centimetre depth but its resolution is hampered by weak signals and high scattering (Fig. 1; (Graves et al., 2004; Lian et al., 2017; Ntziachristos, 2006; Venugopal et al., 2010; Wang et al., 2015; Zacharakis et al., 2011)). The group of photoacoustic techniques including PAT (Dean-Ben et al., 2017; Valluru et al., 2016; Wang and Yao, 2016) and its more refined variants multispectral optoacoustic tomography (MSOT; (Ma et al., 2009; Ntziachristos and Razansky, 2010)) and raster scanning optoacoustic mesoscopy (RSOM; (Omar et al., 2015)) are the newest additions to the optical imaging portfolio. They are special in that light is only used for excitation while sound is what is recorded, thereby rendering them less affected by the shortcomings of using light for imaging thick samples. However, it is important to realize that fundamentally all optical whole-body imaging techniques are severely affected by differential light absorption, scatter and poor depth penetration, precluding full 3D quantification (Fig. 1). Hence, they play a minor role in medical imaging, albeit with a few notable exceptions although outside the field of cell tracking. First, optical coherence tomography (OCT) in ophthalmology (Jung et al., 2011; Tao et al., 2013) and dermatology (Mogensen et al., 2009; Olsen et al., 2015), and, second, photoacoustic imaging as a promising emerging tool in oncology and for the assessment of Crohn’s disease (Diot et al., 2017; Knieling et al., 2017; McNally et al., 2016; Valluru et al., 2016). In summary, despite the combined imaging opportunities provided by the various optical approaches, currently, there is no suitable route for reliable *in vivo* cell tracking available, which requires high sensitivity at good resolution within large fields of view while also providing anatomical context.

## 5 Multi-modal imaging is necessary for *in vivo* cell tracking

For successful *in vivo* cell tracking, it is necessary to build on the strengths of different imaging modalities and combine them with microscopy. CT and MRI both offer anatomical reference, whereby MRI excels in soft-tissue contrast and avoids the use of ionising radiation but is more expensive. The exquisite sensitivity of BLI has been frequently exploited in combination with MRI, *e.g.* for imaging tumour growth or treatment response in preclinical models (Jost et al., 2009; McCann et al., 2009). In animal models, cell tracking by MRI using, for example, iron oxide nanoparticles has been reported, but cross-correlation studies with luciferase/BLI have demonstrated its shortcomings in sensitivity (Song et al., 2009; Zhang et al., 2011). Dual-contrast agents for ^19^F-MRI and fluorescence, *e.g.* perfluorocarbon-TexasRed, have been used to track tumour-associated macrophages in mice (Makela and Foster, 2018). MRI reporter genes have also been developed (Table 1) and have the advantage of co-registration with soft-tissue anatomy and certain functional imaging parameters. However, MRI sensitivity remains poor compared to BLI and radionuclide imaging (Fig. 1). While fluorescent proteins and luciferases are excellent reporters, which also offer multiplexing capability (Mezzanotte et al., 2017; Rodriguez et al., 2017), they suffer from the limitations of optical imaging (see above). In contrast, radionuclide imaging (PET, SPECT) offers best depth penetration and absolute quantification (Lajtos et al., 2014) with preclinical resolutions ≤1 mm (Deleye et al., 2013; Nagy et al., 2013), but radionuclide imaging is more complex to perform and cell detection sensitivities are highly reporter-dependent and cell-specific. Cellular detection sensitivities have been reported to be as good as hundreds/thousands for cancer cells using NIS together with its PET and SPECT radiotracers, respectively, (Diocou et al., 2017; Fruhwirth et al., 2014) and tens of thousands for smaller T-cells using various different reporters in preclinical experiments (Moroz et al., 2015). As PET-CT/MRI and SPECT-CT/MRI instruments are nowadays preclinical and clinical standard, these multimodal approaches offer high sensitivities *via* PET or SPECT combined with CT or MRI, which add anatomical reference at higher resolution than radionuclide imaging techniques (Fig. 1). CLI is unlikely to play a role in *in vivo* cell tracking as it is less sensitive as compared to PET/SPECT and suffering from the shortcomings of optical imaging at depth (see above). Importantly, fluorescence is an excellent partner to complement radionuclide imaging as it excels in the microscopic domain enabling spatial identification of fluorescent cells in tissues (*ex vivo* in tissues or *in vivo* if combined with intravital imaging of specific regions of interest). An additional practical aspect is that genetically encoded fluorescent reporters can be used as selection markers during generation and characterization of radionuclide/fluorescence dual-mode reporter-expressing cells.

## 6 Multi-scale *in vivo* cell tracking in practice

**Fig. 2:**
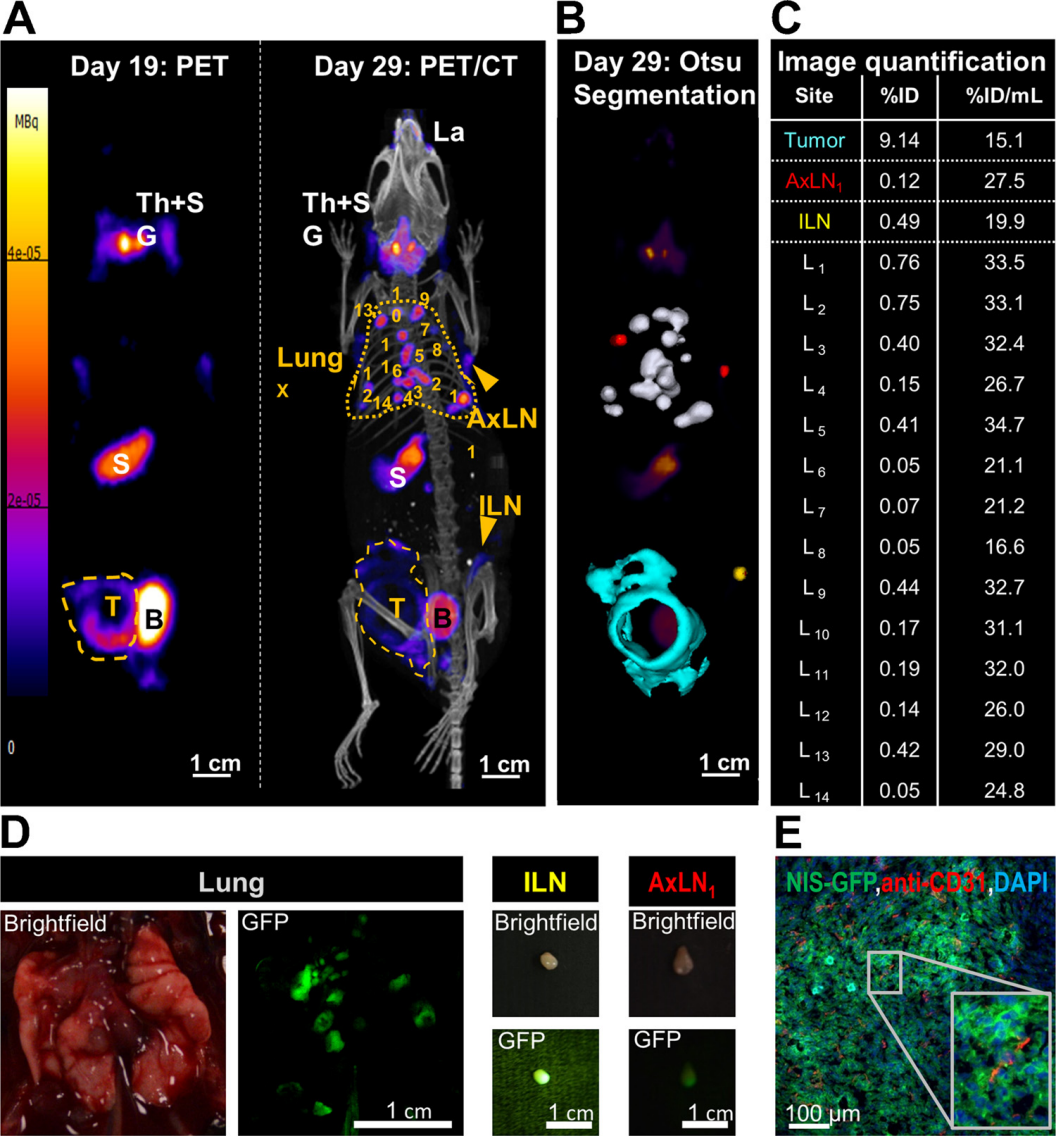
Dual-mode radionuclide-fluorescence metastasis tracking is quantitative and provides data across multiple length scales. Representative results of metastasis tracking in a murine model of inflammatory breast cancer using the radionuclide-fluorescence fusion reporter NIS-GFP are shown. NIS served as an *in vivo* reporter and was imaged by PET/CT using the NIS tracer [^18^F]BF_4_^−^. **(A/left)** On day 19 post tumour inoculation, the primary tumour (yellow dashed line) was clearly identified but no metastasis. It is noteworthy that endogenous NIS signals (white descriptors) were also recorded, *i.e.* the thyroid and salivary glands (Th + SG), the stomach (S), and, at very low levels, some parts of the mammary and lachrymal glands. Neither of these endogenous signals interfered with sites of expected metastasis in this tumour model. The bladder (B) signal stems from tracer excretion. **(A/right)** On day 29 post tumour inoculation, metastases were clearly identified in the lung (yellow dotted line; numbered individual metastases) and in some lymph nodes (inguinal (ILN), axillary (AxLN); yellow arrowheads). The primary tumour (yellow dashed line) had also invaded into the peritoneal wall. Images presented are maximum intensity projections (MIP). **(B)** A 3D implementation of the Otsu thresholding technique enabled 3D surface rendering of cancerous tissues; these are superimposed onto a PET MIP. Lung metastases are shown in white, metastatic axillary lymph nodes in red, the metastatic inguinal lymph node in yellow, and the primary tumour that invaded into the peritoneal wall in turquoise. **(C)** Radiotracer uptake into cancerous tissues was quantified from 3D images (%injected dose (ID)) and normalized by the corresponding volumes (%ID/mL). Individual lung metastases correspond to the numbers in (A). **(D)** NIS-GFP’s fluorescence properties guided animal dissection. As exemplars birghtfield and fluorescence images of the lung with several metastatic lesions and two positive lymph nodes are shown. **(E)** Immunofluorescence histology of the primary tumour. NIS-GFP expressing cancer cells were directly identified without the need for antibody staining. Blood vessels were stained with a rabbit antibody against mouse PECAM-1/CD31 and for nuclei (DAPI) before being imaged by confocal fluorescence microscopy. Data demonstrated vascularization heterogeneity of the primary tumour. The image also shows that the NIS-GFP reporter predominantly resides in the plasma membranes of the tumour cells demonstrating its correct localization to be functional *in vivo* and enabling tumour cell segmentation. (For interpretation of the references to colour in this figure legend, the reader is referred to the web version of this article).

Multi-modal multi-scale imaging has enabled quantitative *in vivo* tracking of tumour growth and spontaneous metastasis in preclinical models. SPECT/CT and PET/CT were used to determine location, organ selectivity and extent of metastasis, while fluorescence streamlined cell line generation and characterization, guided dissection, and enabled straightforward fluorescence histology (Fig. 2) (Diocou et al., 2017; Fruhwirth et al., 2014; Hekman et al., 2017; Minn et al., 2005; Ray et al., 2004; Volpe et al., 2018). Various radionuclide reporters have been used including those offering options to kill administered cells (*e.g.* HSV1-*tk* (Kokoris and Black, 2002; Ponomarev et al., 2004), deoxycytidine kinases (dCK) (Lee et al., 2017; Likar et al., 2010)). Another reporter, NIS, has a long history (Carlson et al., 2009; Che et al., 2005; Dingli et al., 2006; Groot-Wassink et al., 2004; Higuchi et al., 2009; Merron et al., 2007; Sieger et al., 2003; Terrovitis et al., 2008) and excels in cell tracking because it accurately reports cell viability as transport relies on an intact Na^+^/K^+^gradient (Dohan et al., 2003; Portulano et al., 2014). The NIS-fluorescent protein fusion reporter (NIS-FP) (Fruhwirth et al., 2014; Volpe et al., 2018) offers direct accessibility of its subcellular localization (a prerequisite for NIS tracer transport/imaging) at all experimental stages and aids histological tissue segmentation.

[The figure is reproduced with permission and minor rearrangements from (Volpe et al., 2018)].

This approach also enabled imaging how drugs affect tumour progression/metastasis in animal models. For example, etoposide was found to not abrogate spontaneous metastasis in a preclinical model of breast cancer. Metabolic molecular imaging using [^18^F]FDG-PET showed etoposide efficacy in cancer tissues due to etoposide-mediated glucose transporter down-modulation (Witney et al., 2009). But it was NIS-FP that, unaffected by etoposide, enabled quantification of tumour progression in different microenvironments (using serial dual-isotope PET/SPECT/CT imaging). NIS-FP also significantly streamlined the *ex vivo* analysis of etoposide effects on reporter expressing cancer cells (Fruhwirth et al., 2014). Other preclinical radionuclide-fluorescence studies employed, for example, dCK/GFP to investigate tumour growth and Tcell trafficking (Likar et al., 2010), or used SPECT-traceable neural stem cells for glioma targeting (Cheng et al., 2016).

**Fig. 3:**
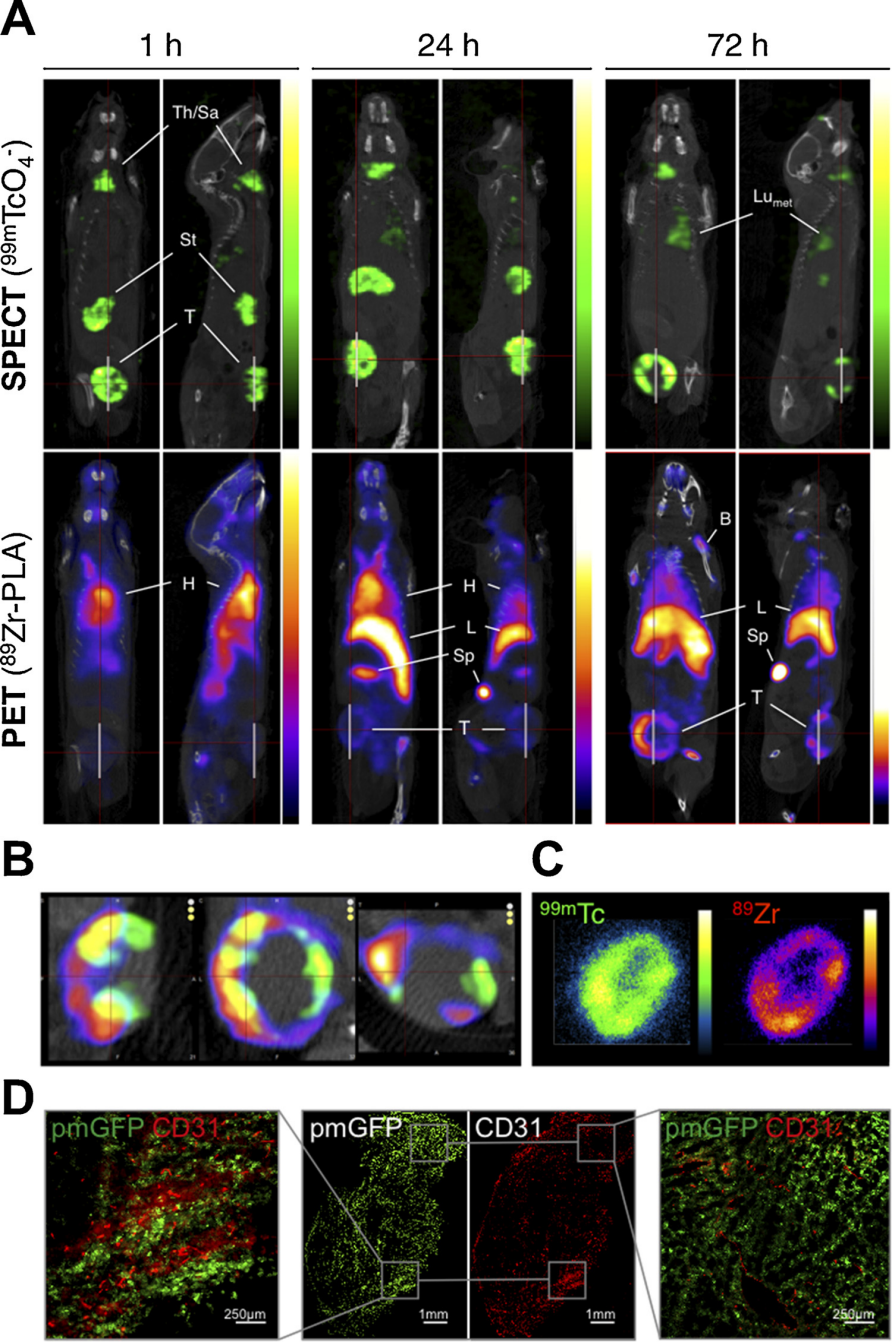
Tracking a nanomedicine to primary and secondary cancer lesions. Liposomal alendronate was radiolabelled with the PET isotope ^89^Zr (^89^Zr-PLA) and administered to animals bearing primary breast tumours that had already spontaneously metastasized (as determined by ^99m^TcO4^−^-afforded NIS-SPECT/CT). **(A)** Coronal and sagittal SPECT-CT (top; cancer cells) and PET-CT (bottom; nanomedicine) images centred at the tumours of the same animal are shown at indicated time points after intravenous administration of ^89^Zr-PLA. SPECT-CT images show identical biodistribution over time with high uptake in endogenous NIS-expressing organs (stomach, thyroid) and NIS-FP-expressing cancer cells in the primary tumour (T) and metastases (LN_met_ and Lu_met_). PET-CT images show the increasing uptake of ^89^Zr-PLA over time in the primary tumour (T), spleen (Sp), liver (L), and bone (B) and decreasing amounts in the blood pool/heart (H). For corresponding time–activity curves refer to (Edmonds et al., 2016). **(B)** Co-registered SPECT/PET/CT images of the primary tumour (from left to right: sagittal, coronal, transverse) showing a high degree of colocalization but also intra-tumoral heterogeneity of ^89^Zr-PLA (purple scale); ^99m^TcO_4_^−^NIS signals (green scale) show live cancer cells. **(C)** Autoradiography images (left, ^99m^Tc; right, ^89^Zr) of a coronal slice from the same tumour as in (B) showing a high degree of colocalization and heterogeneity. **(D)** Fluorescence microscopy of an adjacent slice of the same tumour as in (B/C) showing areas of high and low microvascular density (determined by anti-CD31 staining). (For interpretation of the references to colour in this figure legend, the reader is referred to the web version of this article).

Multiplex imaging also enabled differential tracking of molecular and cellular therapeutics to cancer tissues in animal models. In luciferase-expressing non-small cell lung cancers gadolinium- and Cy5.5-labelled nanoparticles were evaluated as potential orotracheally administered tumour diagnostics. Tumour cell tracking relied on BLI while MRI visualized the diagnostic agent and provided anatomical reference, and fluorescence streamlined histological confirmation (Bianchi et al., 2014). Combined serial PET/SPECT/CT-fluorescence imaging also enabled tracking of radiolabelled liposomal mevalonate pathway inhibitors to NIS-FP-expressing tumours and metastases (Fig. 3). This study demonstrated the need for a longer interval between administration of this γδ Tcell therapy booster (Lavoue et al., 2012; Mattarollo et al., 2007; Parente-Pereira et al., 2014) and the corresponding γδ Tcell therapy (Edmonds et al., 2016). Conventional reporter gene-based tracking of adoptive cell therapies has also been performed (Koya et al., 2010; Likar et al., 2010; Moroz et al., 2015). The full potential of co-tracking the cell therapy to the tumour was unlocked only very recently; by co-tracking PET-traceable γδ Tcells to NIS-FP-traceable cancer cells in an animal model of human breast cancer, demonstrating that liposomal alendronate pre-treatment caused higher tumour uptake of γδ Tcells (Man et al., 2017). Notably, also as a proof-of-principle study in human glioma patients has recently been performed, employing PET for intra-organ administered CART tracking with MRI providing anatomical context (Keu et al., 2017).

[The figure is reproduced from (Edmonds et al., 2016) (https://pubs.acs.org/doi/10.1021/acsnano.6b05935) with permission from ACS; further permissions related to the material excerpted should be directed to the ACS].

## 7 Conclusion and outlook

Multi-modal multi-scale *in vivo* cell tracking is a rapidly growing interdisciplinary area, which has been fuelled by the rise of cell-based therapies and enabled by recent technological advances providing enhanced resolution, sensitivity and multiplexing capabilities on both the macroscopic and microscopic scales. For long-term *in vivo* cancer cell tracking, reporter gene methodologies are particularly attractive. The most promising methodologies to-date exploit the exquisite sensitivity, multiplex capability and 3D quantification of radionuclide imaging and combine them with fluorescence methodologies, thereby allowing convenient cell line generation and reliable and versatile *ex vivo* microscopic analyses. *In vivo* cell tracking cannot always be directly translated for human use because fluorescent proteins, luciferases and certain non-human radionuclide reporters have no direct clinical utility. But importantly, preclinical *in vivo* cell tracking serves as a versatile platform for understanding the underlying biology and to validate therapeutic concepts, thereby informing subsequent clinical trials. However, in the case of live cell therapies, *in vivo* cell tracking provides the means for long-term monitoring in patients if required. It is noteworthy that cell therapies are emerging also in other fields than cancer including transplantation immunology (Boardman et al., 2017) and regenerative medicine (Ellison et al., 2013; Rashid et al., 2015). Multi-modal multi-scale *in vivo* imaging-afforded cell tracking is therefore likely to become increasingly important for the successful development of such cell therapies, particularly in the context of therapy safety and monitoring.

## Conflict of interest statemment

The authors declare that they have no competing financial interests.
